# Device-related events do not alter long-term psychological distress after implantable cardioverter-defibrillator implantation: A 24-month prospective cohort study

**DOI:** 10.1016/j.hroo.2026.04.004

**Published:** 2026-04-11

**Authors:** Marthe J. Huntelaar, Anne-Lotte C.J. van der Lingen, Mischa T. Rijnierse, Saskia Elshout, Vokko P. van Halm, Anna E. Leeuwis, Luuk H.G.A. Hopman, Cornelis P. Allaart

**Affiliations:** 1 Department of Cardiology, Amsterdam Cardiovascular, Amsterdam UMC, Vrije Universiteit Amsterdam, Amsterdam, The Netherlands; 2 Department of Internal Medicine, Onze Lieve Vrouwe Gasthuis, Amsterdam, The Netherlands; 3 Department of Cardiology, Jeroen Bosch Ziekenhuis, ’s-Hertogenbosch, The Netherlands; 4 Department of Cardiology, Spaarne Gasthuis, Haarlem, The Netherlands; 5 Alzheimer Center and Department of Neurology, Amsterdam UMC, Vrije Universiteit Amsterdam, Amsterdam, The Netherlands; 6 Department of Medical Psychology, Amsterdam UMC, Vrije Universiteit Amsterdam, Amsterdam, The Netherlands; 7 Department of Cardiology, Radboud UMC, Radboud Universiteit, Nijmegen, The Netherlands

**Keywords:** Implantable cardioverter-defibrillator, Psychological distress, Device-related events, Florida Shock Anxiety Scale, ICD therapy events, ICD complications

## Abstract

**Background:**

Implantable cardioverter-defibrillators (ICDs) have long been established as effective in reducing all-cause mortality in patients at risk of sudden cardiac death. However, in recent years, the balance between benefit and harm has come under scrutiny. A substantial proportion of patients with an ICD experience psychological distress.

**Objective:**

This study aimed to evaluate whether device-related events influence psychological outcomes after ICD implantation.

**Methods:**

The prospective PSYCHE-ICD study enrolled 178 patients (mean age 64 ± 12 years; 79% male) undergoing ICD or cardiac resynchronization therapy defibrillator implantation with a follow-up time of 24 months. Device-related events, including complications and ICD therapy events, were assessed. Psychological outcomes were measured using the Florida Shock Anxiety Scale, Inventory of Depressive Symptomatology, and Beck Anxiety Inventory at baseline and 2, 12, and 24 months after ICD implantation.

**Results:**

Device-related events occurred in 33% of patients. Over 24 months, Florida Shock Anxiety Scale, Inventory of Depressive Symptomatology, and Beck Anxiety Inventory scores all decreased significantly (all *P* < .005), indicating an overall reduction in psychological distress, independent of device-related events. Psychological distress at 24 months was primarily predicted by baseline depressive and anxiety symptoms, but not by the occurrence of device-related events.

**Conclusion:**

Psychological distress generally decreases during the first 24 months after ICD implantation. Long-term outcomes are determined more by pre-existing psychological vulnerability than by the occurrence of device-related events.

## Graphical abstract

**Figure:**
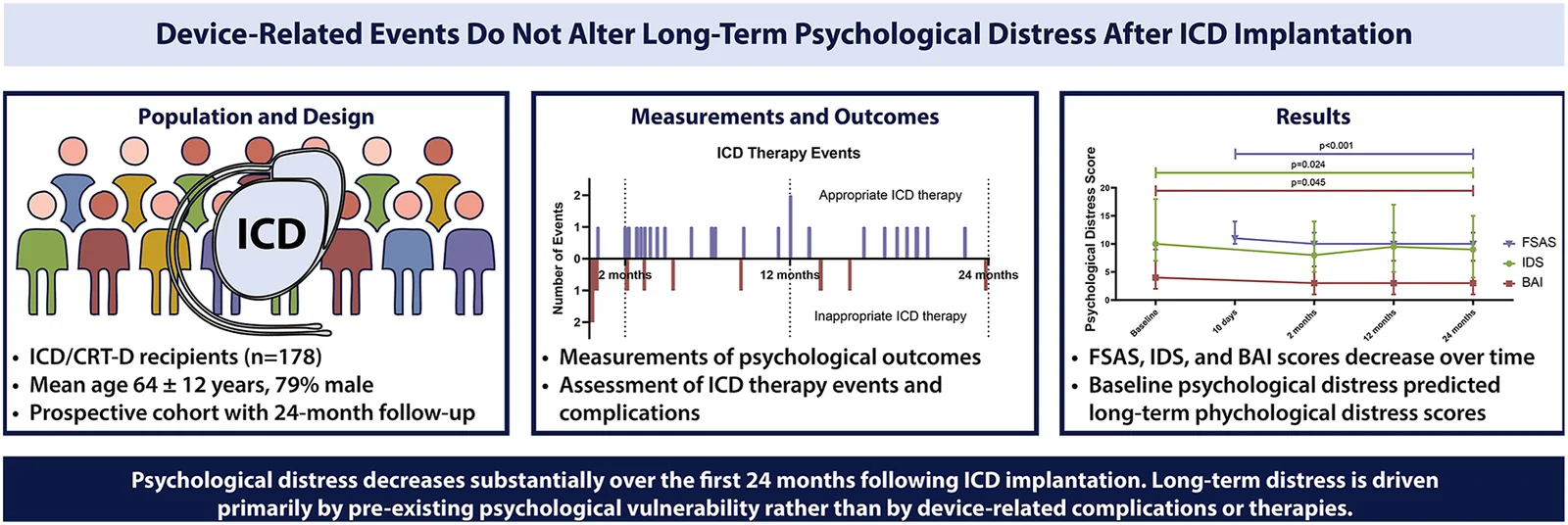

**Key Findings**

▪ Psychological distress decreased significantly over the 24 months after implantable cardioverter-defibrillator implantation.
▪ Although one-third of patients experienced device-related events, these events were not associated with adverse long-term psychological outcomes.
▪ Baseline depressive and anxiety symptoms were the strongest predictors of psychological distress at 24-month follow-up.

## Introduction

Implantable cardioverter-defibrillators (ICDs) have long been established as effective in reducing all-cause mortality in patients at risk of sudden cardiac death. However, in recent years, the balance between benefit and risk has come under increasing scrutiny, particularly in the context of primary prevention ICD implantation.^1^, ^2^, ^3^ Advances in pharmacologic therapy and improvements in imaging and genetic risk stratification, along with concerns about device-related adverse events, have prompted more nuanced guideline recommendations.^4^ In this shifting balance between benefit and harm, outcomes such as quality of life and psychological well-being are gaining importance in the indication process.

A substantial proportion of patients may experience psychological distress manifesting as fear of shocks, anxiety, or depressive symptoms, which often persists long after device implementation.^5^, ^6^, ^7^ The development of psychological distress after ICD implantation is considered multifactorial. Although the burden of underlying cardiac disease and the need for further interventions may contribute, distress is often amplified by device-related complications or therapeutic interventions, particularly appropriate and inappropriate ICD shocks.^8^, ^9^, ^10^, ^11^ These events not only increase morbidity and mortality but may also have a profound psychological impact, an aspect that remains insufficiently explored.^9^^,^^10^^,^^12^^,^^13^ Beyond clinical events, psychological vulnerability further shapes individual responses. In particular, type D personality, characterized by negative affectivity and social inhibition, has consistently been linked to heightened emotional reactivity and poorer psychological adjustment after ICD implantation.^14^

Despite growing recognition of these associations and in the light of shifting indications for ICDs, few studies have prospectively examined the interplay between device-related events and longitudinal psychological outcomes. Addressing this gap is needed to inform future guideline recommendations and enhance psychosocial care and patient counseling. In this prospective cohort study, we examined whether device-related events, including complications and ICD therapy events, were associated with increased psychological distress. The primary objective was to determine whether fear of shock increases after such events and assess whether these effects are transient.

## Methods

### Study design

The PSYCHE-ICD study is a single-center, prospective observational cohort study conducted at the Amsterdam University Medical Center (Amsterdam UMC), The Netherlands. A detailed description of the PSYCHE-ICD study design and the baseline results have been published previously.^6^ Eligible patients included those undergoing ICD implantation for either primary or secondary prevention, with or without cardiac resynchronization therapy defibrillator (CRT-D). There were no exclusion criteria based on the etiology of cardiomyopathy. Patients were excluded if they had insufficient proficiency in the Dutch language or if complete follow-up was deemed unlikely. A history of psychiatric or psychological conditions was explicitly not an exclusion criterion. The study protocol was approved by the institutional medical ethics committee of the Amsterdam UMC and adhered to the Declaration of Helsinki. All participants provided oral and written informed consent.

### Baseline characteristics

Baseline demographic and clinical characteristics, New York Heart Association class, comorbidities, medication use, and baseline psychological assessment were collected 1 day before device implantation.

Patients received an ICD or CRT-D according to the European Society of Cardiology guidelines.^15^ Devices were programmed per protocol based on the most recent available scientific evidence. At the time of implantation, devices were programmed with detection zones for ventricular arrhythmias using the following cutoffs: >150 beats per minute (bpm) (monitor zone), >180 bpm (ventricular tachycardia [VT] 1 zone), and >250 bpm (ventricular fibrillation [VF] zone) with extended detection intervals. Programming was individualized when clinically indicated. Routine device interrogations were scheduled at 10 days, 2 months, 12 months, and 24 months. All patients were enrolled in remote monitoring, with data reviewed by certified device technicians and electrophysiologists.

### Device-related events

Device-related events were classified into 2 categories: complications (categorized as major or minor) and device therapy events (ie, appropriate or inappropriate antitachycardia pacing [ATP] and/or ICD shocks). Major complications included lead dislodgement, pneumothorax, endocarditis or lead infections, replacements, and VT ablation. The latter 2 were included in this category because it reflects an event indicating failure of ICD therapy and adjunctive medical treatment to adequately control ventricular arrhythmias. For each predefined follow-up interval (baseline to 2 months, 2–12 months, and 12–24 months), the occurrence of any device-related event was assessed. Events were evaluated per interval, with each period treated independently; if an event occurred in 1 interval, the next interval was considered event-free unless a new event was recorded. Appropriate device therapy was defined as ATP or shock delivered for sustained VT or VF. Inappropriate ICD therapy was defined as ATP or shock delivered for any rhythm other than VT or VF.

### Fear of shocks and psychological well-being

For assessment of the level of fear of shocks, the validated Florida Shock Anxiety Scale (FSAS) was used.^16^ This questionnaire was first administered 10 days after implantation and subsequently 2, 12, and 24 months after implantation. The FSAS is a 10-item questionnaire designed to measure the level of anxiety owing to the presence of the ICD and the possibility of receiving an ICD shock. It is rated on a 5-point Likert scale (1 = not at all up to 5 = all the time), with higher scores indicating greater fear. The cutoff value was based on the population median.

Psychological distress was assessed at 4 time points: 1 day before implantation (baseline) and at 2, 12, and 24 months after implantation. Depression symptoms were measured using the 30-item Inventory of Depressive Symptomatology (IDS)^17^ with each item scored on a 4-point Likert scale (0–3). A total score of ≥14 was considered indicative of depressive symptoms. Anxiety symptoms were assessed using the Beck Anxiety Inventory (BAI),^18^ a 21-item questionnaire using a 4-point Likert scale (0 = not at all to 3 = severely). Scores of ≥8 were considered indicative of clinically relevant anxiety.

Presence of type D personality was assessed at baseline using the 14-item Type D Scale.^19^ This evaluates 2 personality traits: negative affectivity and social inhibition. Each subscale comprises 7 items rated on a 5-point Likert scale (0 = false up to 4 = true). A type D personality is defined by scores of ≥10 on both subscales.

### Outcomes

The primary outcome was the association between device-related events and shock-related anxiety over 24 months, as measured by FSAS. Secondary outcomes included the association between device-related events and symptoms of anxiety and depression. The types of psychotropic medication used and their indications were highly heterogeneous, precluding detailed statistical analysis of their effects on outcomes. Exploratory analyses examined the relationship between type D personality and the experience of phantom shocks. Phantom shocks were defined as patient-reported sensations perceived as an ICD shock in the absence of any device-recorded therapy or arrhythmic event upon interrogation.^20^

### Statistical analysis

Continuous variables are presented as mean ± standard deviation or median (25th to 75th percentile) depending on the distribution. Categorical variables are expressed as absolute number (percentage). Differences in baseline characteristics between groups (eg, patients with vs without device-related events) were analyzed using the independent samples *t* test or Mann-Whitney U test for continuous variables and χ^2^ or Fisher’s exact test for categorical variables.

Longitudinal analysis of psychological distress (FSAS, IDS, BAI) is performed using Wilcoxon signed rank tests. Cross-sectional analysis of differences in psychological distress between patients with and without an event during a certain period is performed using Mann-Whitney U tests.

Forward multivariate logistic regression is used to determine which variables are potential predictors of psychological distress 24 months after implantation of the ICD. Stratified Kaplan-Meier analysis is performed to determine whether patients with type D personality are more prone to developing device-related events during 24-month follow-up.

For every patient, questionnaires with >10% missing data were excluded. Missing data with <10% of the data missing are imputed by the mean of the regarding questionnaire.

A 2-tailed *P* < .05 is considered statistically significant. Statistical analyses are conducted using SPSS (version 28).

## Results

### Patient characteristics

A total of 178 patients were included in the study (Table 1). The mean age was 64 ± 12 years, and 141 patients (79%) were male. At baseline, 49 patients (28%) received a CRT-D. Type D personality was identified in 35 patients (20%), 62 patients (35%) had depressive symptoms, and 55 (31%) had anxiety symptoms.

**Table 1:** Baseline characteristics for the total population and between patients with and without device-related events

Characteristics	Total cohort (n = 178)	Device-relatedevent in 2 y (n = 59)	No device-relatedevent in 2 y (n = 119)	*P* value
Demographics				
Age (y)	64 ± 12	65 ± 10	64 ± 12	.337
Male sex	141 (79%)	46 (78%)	95 (80%)	.458
Cardiac risk factors				
Former/current smoker	134 (75%)	45 (76%)	89 (75%)	.868
Stroke	24 (13%)	7 (12%)	17 (14%)	.424
Hypertension	85 (49%)	27 (46%)	58 (49%)	.415
Diabetes mellitus	40 (23%)	13 (22%)	27 (23%)	.514
BMI (kg/m^2^)	27 ± 4	28 ± 5	27 ± 4	.509
Cardiac history				
NYHA class II–IV	114 (64%)	36 (61%)	78 (66%)	.333
Ischemic disease	107 (60%)	36 (61%)	71 (60%)	.497
Cardiomyopathy				.692
Ischemic	80 (45%)	25 (42%)	55 (46%)	
Nonischemic	81 (45%)	23 (30%)	58 (49%)	
OHCA	42 (24%)	18 (31%)	24 (20%)	.091
Atrial fibrillation	62 (35%)	27 (46%)	35 (30%)	.024∗
LVEF (%)	35 ± 13	34 ± 14	34 ±1 2	.959
QRS duration (ms)	123 ± 31	126 ±34	123 ±31	.546
Psychiatric history				
History of psychiatric problems	32 (18%)	12 (20%)	20 (17%)	.681
Device indication				
Primary prevention	128 (72%)	36 (61%)	92 (77%)	.019∗
Device type				
CRT-D	49 (28%)	15 (25%)	34 (29%)	.685
Medication at discharge after implantation				
ACEi/ARB	150 (84%)	36 (61%)	83 (70%)	.160
		43 (73%)	19 (16%)	
Beta-blockers	148 (83%)	43 (73%)	105 (88%)	.010∗
Diuretics	89 (50%)	31 (53%)	58 (49%)	.375
Psychotropic agents	44 (24%)	21 (36%)	23 (19%)	.016∗
Laboratory values				
Creatinine (μmol/L)	93 (57–129)	94 (76–122)	91 (77–108)	.472
NT-proBNP (ng/L)	888 (369–2075)	790 (368–1995)	933 (368–2260)	.749
Questionnaires TypeD	35 (20%)	14 (24%)	21 (18%)	.221
Depressive symptoms (IDS ≥14)	62 (35%)	27 (46%)	35 (30%)	.040∗
Anxiety symptoms (BAI ≥8)	55 (31%)	20 (34%)	35 (30%)	.369
IDS score	10 (7–18)	12 (6–25)	10 (7–17)	.144
BAI score	4 (2–9)	4 (1–13)	4 (2- 9)	.994

ACEi = angiotensin-converting enzyme inhibitor; ARB = angiotensin receptor blocker; BAI = Beck Anxiety Inventory; BMI = body mass index; CRT-D = cardiac resynchronization therapy defibrillator; IDS = Inventory of Depressive Symptomatology; LVEF = left ventricular ejection fraction; NT-proBNP = N-terminal pro-brain natriuretic peptide; NYHA = New York Heart Association; OHCA = out-of-hospital cardiac arrest; TypeD = type D personality.

∗ Statistically significant.

During the 24-month follow-up, 59 patients (33%) experienced at least 1 device-related event, that is, complication or ICD therapy. These patients had more depressive symptoms at baseline (46% vs 30%; *P* = .040), higher baseline psychotropic medication use (36% vs 19%; *P* = .016), and higher rates of atrial fibrillation (46% vs 30%; *P* = .034) than patients without device-related events. Patients with device-related events also more often received an ICD for secondary prevention (39% vs 23%; *P* = .019) and less frequently used beta-blockers (73% vs 88%; *P* = .010). No differences were observed in sex, underlying cardiomyopathy, or cardiovascular risk profile.

### Device-related events

In the 59 patients who experienced a device-related event, complications occurred in 29 patients (16%), of which 20 (11%) were classified as major complications (Table 2). 5 patients experienced 2 complications, and 1 patient had 3.

**Table 2:** Frequency of the different events.

Device-related events, n = 59 (33%)	Type	Complication or event	Number (percentage)
Complications, n = 29 (16%)	Major, n = 20 (11%)	Early lead dislodgement	4 (2)
		Late lead dislodgement	4 (2)
		Pneumothorax	2 (1)
		Endocarditis/lead infection	1 (1)
		Replacement/upgrade	5 (3)
		VT ablation	8 (4)
	Minor, n = 9 (5%)	Hematoma	3 (2)
		Thrombosis	2 (1)
		Wound infect	2 (1)
		Unsuccessful DFT	3 (2)
		Inadequate sensing	2 (1)
ICD therapy events, n = 43 (24%)	Appropriate, n = 29 (16%)	ATP only	17
		Shock∗	12
		ATP followed by shock	6
	Inappropriate, n = 15 (8%)	ATP only	3
		Shock∗	12
		ATP followed by shock	6
Phantom shocks, n = 7 (4%)		Phantom shock	7 (4)

ATP = antitachycardia pacing; DFT = defibrillation threshold test; ICD = implantable cardioverter-defibrillator; VT = ventricular tachycardia.

∗ Shock may be preceded by ATP.

**Figure 1:**
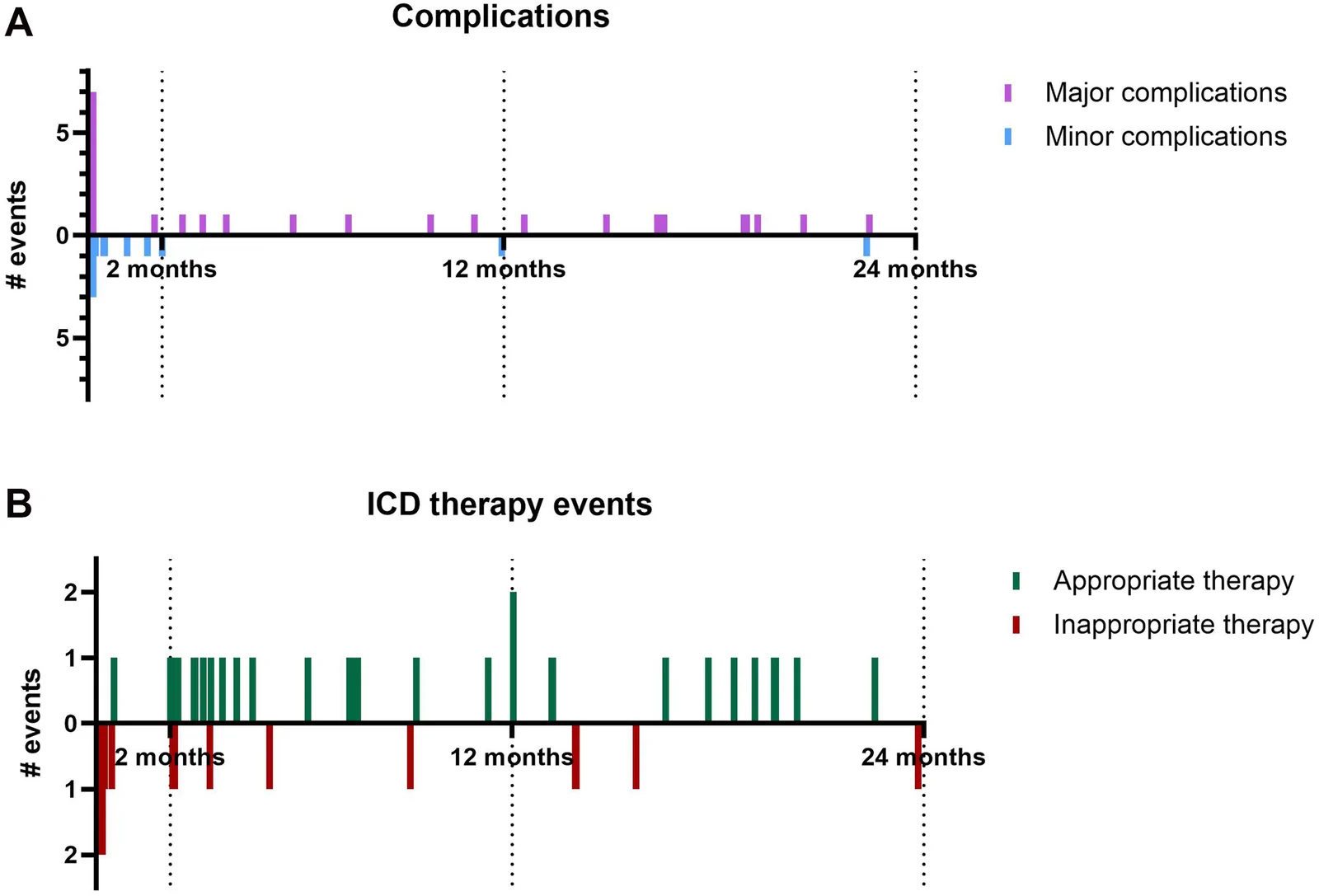
Temporal distribution of complications (**A**) and ICD therapy events (**B**) over 24 months of follow-up. ICD = implantable cardioverter-defibrillator.

ICD therapy was delivered in 43 patients (24%), of whom 29 (16%) experienced appropriate ICD therapy and 15 (8%) experienced inappropriate ICD therapy. 1 patient experienced both appropriate and inappropriate ICD therapy. The distribution of therapies, further specified by ATP and shock, is presented in Table 2. Temporal distribution of complications, ICD therapy events, and device-related complications is presented in Figure 1.

### Fear of shock

**Figure 2:**
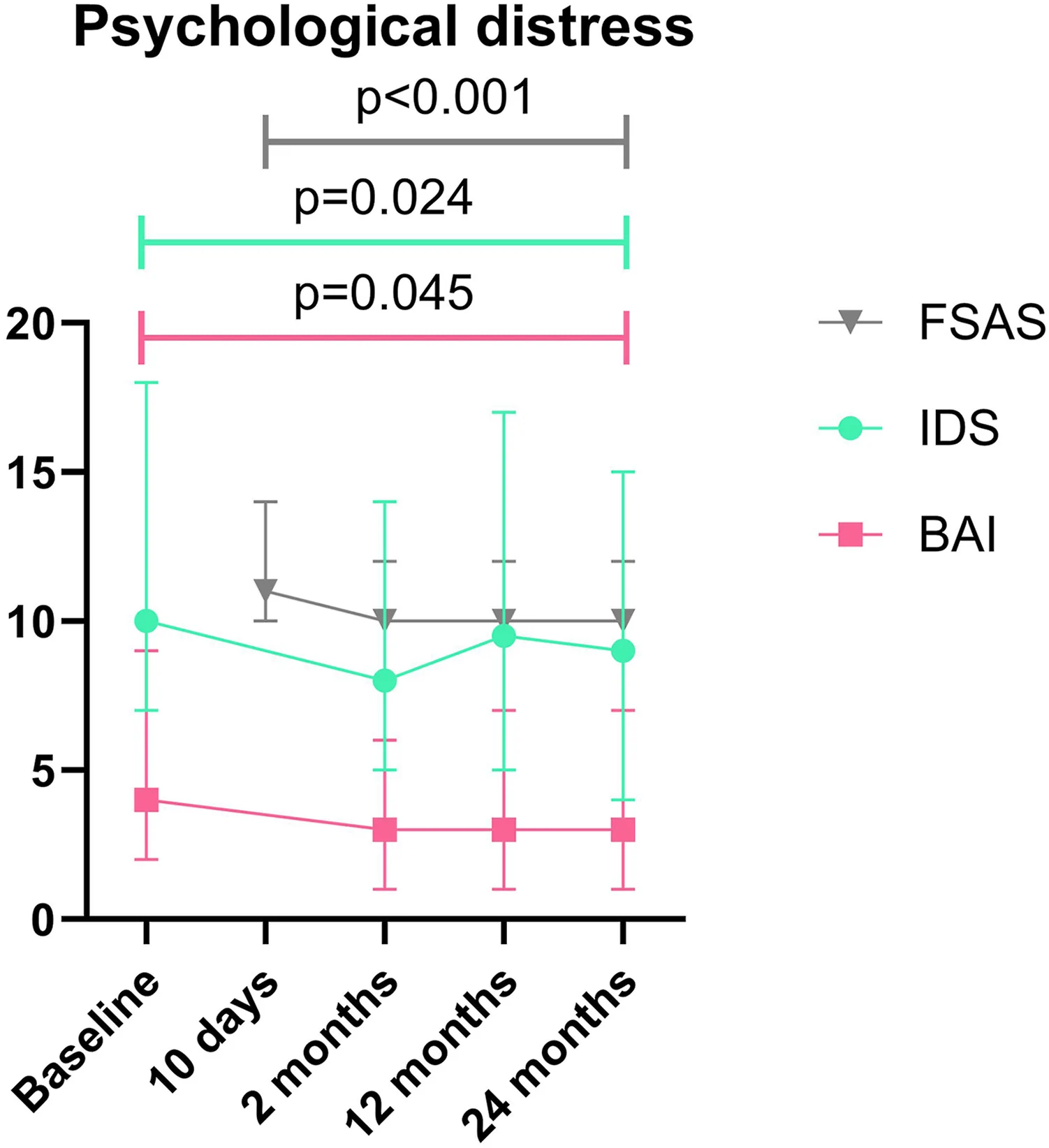
IDS, BAI, and FSAS score of the total population over time. IDS = Inventory of Depressive Symptomatology; BAI = Beck Anxiety Inventory; FSAS = Florida Shock Anxiety Scale.

**Figure 3:**
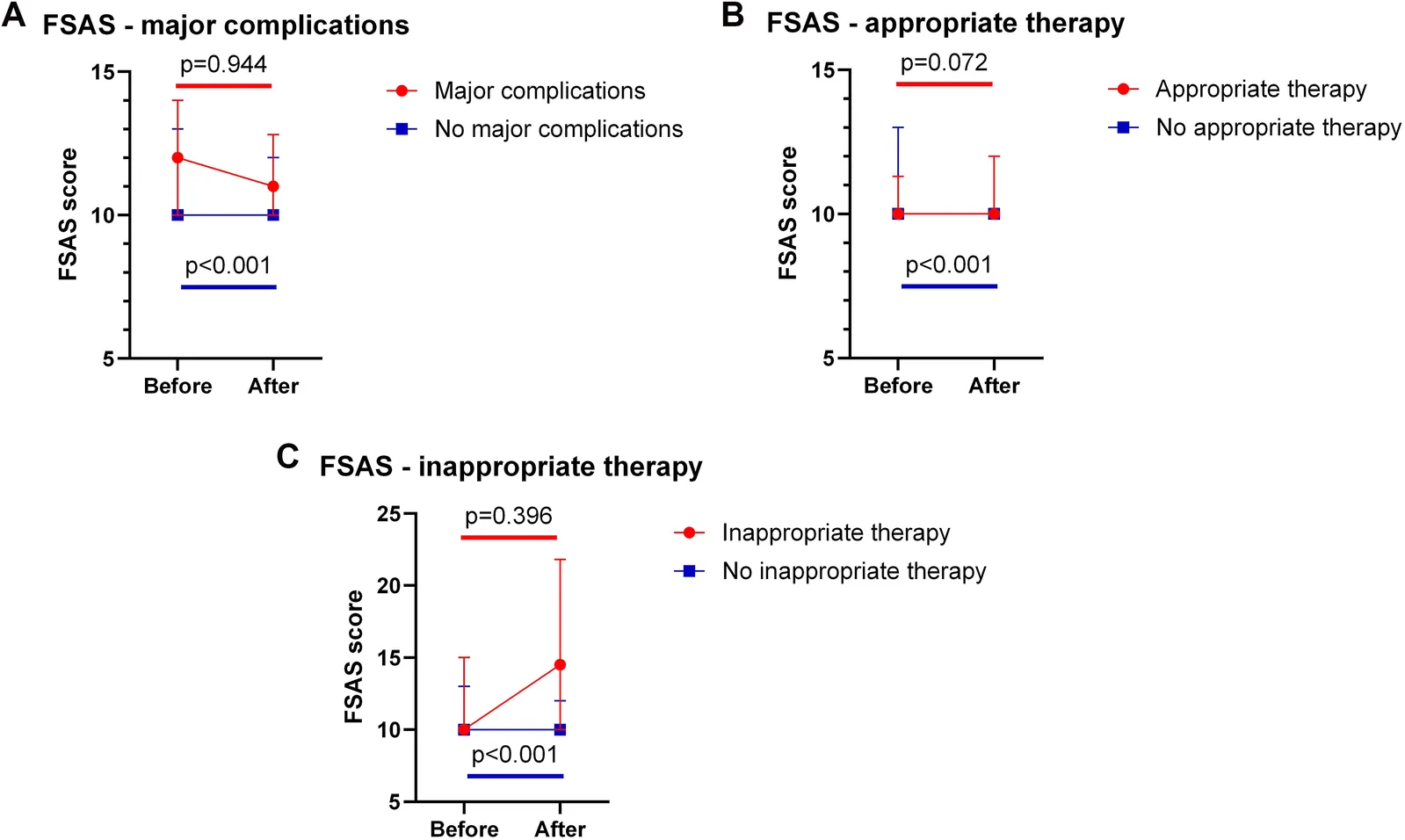
FSAS scores before and after (**A**) major complications, (**B**) appropriate therapy, and (**C**) inappropriate therapy. FSAS = Florida Shock Anxiety Scale.

Median FSAS scores decreased significantly in the total cohort from baseline to 24 months (11 (10–14) to 10 (10–12); *P* < .001), with the greatest reduction observed after the first 2 months (11 (10–14) to 10 (10–12); *P* < .001) (Figure 2, Supplemental Table 1). No significant differences were observed between patients with and without complications (major or minor) or ICD therapy events (appropriate or inappropriate) during follow-up (Supplemental Figure 1, Supplemental Table 2). FSAS scores at baseline and after 24 months, grouped by the occurrence of the different device-related events, did decrease significantly in patients without device-related events, major complications, appropriate therapy, and inappropriate therapy. However, a discrepancy is observed between temporary and long-term effects. When comparing FSAS scores before and after an event, FSAS scores are increased after a ICD therapy event, although nonsignificant, whereas FSAS scores decreased significantly in the patients without a therapy event, as presented in Figure 3.

In multivariate logistic regression, FSAS of >10 (median of the population) at 24 months was independently associated with younger age (odds ratio [OR] = 0.930; *P* = .002), higher baseline depressive symptoms (OR 4.437; *P* = .026), and higher baseline anxiety symptoms (OR 4.421; *P* = .026). When baseline depressive and anxiety symptoms were excluded from the model, a history of out-of-hospital cardiac arrest predicted elevated FSAS scores (OR 3.333; *P* = .015).

### Depressive and anxiety symptoms

Median scores of depressive and anxiety symptoms also declined significantly from baseline to 24 months (Figure 2, Supplemental Table 1). At 24 months, there were no significant differences in IDS or BAI scores between patients with and without device-related events (Supplemental Tables 3 and 4). Neither complications, whether major or minor, nor ICD therapy events, either appropriate or inappropriate, were associated with significant differences in depression or anxiety symptom trajectories. Although CRT typically improves functional capacity and quality of life in responders,^21^ we did not observe significant differences in FSAS, IDS, or BAI scores between ICD and CRT-D recipients in our exploratory analysis (Supplemental Table 5).

In multivariate logistic regression, the presence of depressive and anxiety symptoms at 24 months was predicted by several factors, such as depressive symptoms at baseline (Table 3), but not device-related events.

**Table 3:** Multivariate logistic regression for the presence of depressive symptoms after 24 months of follow-up and the presence of anxiety symptoms after 24 months of follow-up.

Multivariate logistic regression	Including baseline depressive and anxiety symptoms	
	Odds ratio	*P* value
Depressive symptoms at 24 mo		
Diabetes	10.67	.073
Type D personality	9.16	.035
Baseline depressive symptoms	36.89	.004
Anxiety symptoms at 24 mo		
Atrial fibrillation	0.08	.021
6-minute walking distance	1.01	.056
Nonsustained VT	0.12	.028
Baseline depressive symptoms	12.26	.005
Baseline anxiety symptoms	14.56	.003

VT = ventricular tachycardia.

IDS scores at 24 months were not available for all participants (n = 60). Comparative analysis showed no significant differences in median IDS scores at baseline (10 [9–15] vs 11 [9–14]; *P* = .684) nor in the proportion of patients with depressive symptoms (39% vs 34%; *P* = .577) between patients with and without follow-up data. This suggests that IDS scores at 24 months were missing at random.

### Phantom shocks and type D personality

Phantom shocks were reported in 7 patients (4%), of whom 5 (71%) had type D personality. A significant correlation was observed between phantom shocks and type D personality (Spearman’s ⍴ = 0.261; 95% confidence interval 0.112–0.299; *P* < .001). Phantom shocks were not significantly correlated with baseline depressive or anxiety scores. Kaplan-Meier analysis showed no significant difference in device-related event rates between patients with and without type D personality (log-rank *P* = .278).

## Discussion

In this prospective observational study, the impact of device-related complications and device therapy events was evaluated over 24 months after ICD implantation. Shock anxiety as determined by the FSAS score declined over 24 months, independent of the occurrence of device-related events. Similarly, depressive symptoms and anxiety scores declined during follow-up independent of the occurrence of device-related events. Baseline depressive and anxious symptoms predicted the presence of depressive and anxiety symptoms 24 months after ICD implantation. These findings suggest a general trend toward psychological adaptation after ICD implantation and device-related events and that long-term psychological distress is more strongly influenced by baseline depressive symptoms and anxiety than by device-related events.

### Occurrence of ICD-related events

In total, 33% of the patients in the cohort experienced a device-related event. Device-related complications occurred in 16% of our cohort, with 11% classified as major. These rates are within the range reported in previous studies, despite variations in definitions and follow-up.^22^, ^23^, ^24^ It should be noted that device upgrades and VT ablation were included among the major complications. This categorization was chosen because ICD therapy and antiarrhythmic medication are intended to prevent the need for VT ablation or upgrade; therefore, VT ablation and upgrades were considered to reflect clinically relevant ICD failure.

Rates of appropriate (16%) and inappropriate therapy (8%) were consistent with contemporary ICD cohorts, in which 10%–20% of the patients receive appropriate therapy within 2 years, depending on patient selection and programming settings, and fewer than 10% per year experience inappropriate therapy under modern programming strategies.^22^^,^^25^^,^^26^

### Fear of shock after ICD implantation

The FSAS was originally validated as a tool to assess ICD shock-related anxiety. However, subsequent studies have expanded its application to encompass broader constructs of device-related anxiety, acknowledging that factors beyond shocks, such as device dependence, complications, and lifestyle limitations, can contribute to distress.^27^ In our study, device-related events were not independently associated with persistent psychological distress at 24 months after ICD implantation. Consistent with the findings of Morken et al,^28^ who reported higher fear of shock among patients with a history of shocks or recent tachyarrhythmia events (<12 months), but otherwise generally low levels of shock-related anxiety, we observed that fear of shock did not persist at 2-year follow-up. The absence of long-term associations between device-related events and fear of shock may reflect transient psychological responses^29^, ^30^, ^31^ or suggest a dose–response relationship, in which multiple therapies are required to sustain heightened shock anxiety.^12^^,^^32^ The systematic review by da Silva^30^ on quality of life in patients with ICDs highlights the heterogeneous effects of ICD implantation, suggesting that both timing of assessments and study design play an important role in the observed outcomes. In our study, the outcomes may have been influenced by the varying time interval between the device-related events and subsequent psychological assessments. In addition, the small number of patients with multiple shocks (n = 9; 4 appropriate and 5 inappropriate) precluded formal analysis.

In our cohort, FSAS changes were more pronounced after inappropriate therapy than after major complications, likely reflecting the scale’s emphasis on shock-related concerns. In contrast, major complications, being heterogeneous in nature, likely influence patient perceptions and anxiety through multiple mechanisms beyond shock.

### Symptoms of depression and anxiety after ICD implantation

Depressive and anxiety symptoms reduced over 24 months and were not significantly influenced by device-related events. This suggests that the occurrence of a device-related event alone is insufficient to predict long-term psychological outcomes. Our findings further demonstrate that psychological distress declined between baseline and 24 months, in line with previous literature^33^^,^^34^ and with comparable levels at follow-up in patients who did and did not experience device therapy. In contrast, poorer baseline psychological status consistently predicted poorer long-term outcomes. Pre-implantation depressive and anxiety symptoms were among the strongest and most consistent predictors of persistent fear of shock, anxiety, and depressive symptoms at 24 months, in line with previous studies.^9^^,^^19^ The literature further identifies additional risk factors for heightened fear of shocks, including insufficient knowledge about the device, low perceived provider support, posttraumatic stress symptoms, and negative beliefs regarding device dependence.^27^ Taken together, these findings emphasize the importance of pre-implantation screening to identify high-risk patients, who may particularly benefit from tailored education and psychosocial support interventions.^31^^,^^35^, ^36^, ^37^

### Phantom shocks and type D personality

Phantom shocks were reported by 4% of our patients, a prevalence similar to previous findings of 5.4% in a cohort of 300 patients.^38^ In contrast to earlier studies, phantom shocks in our population were associated with type D personality, whereas no correlations were found with symptoms of depression and anxiety.^20^ The concept of type D personality should be interpreted with caution. Dichotomizing inherently continuous personality traits may inflate observed effects.^39^ Nevertheless, type D personality has consistently been linked to adverse psychological and clinical outcomes in cardiac populations. Our findings suggest that integration of psychological screening and additional brief interventions or targeted support into multidisciplinary ICD care may enhance patient well-being, even if psychological effects after ICD implantation, device-related events, or phantom shocks are typically transient.

### Limitations

Several limitations of this research should be acknowledged. First, baseline psychological distress was measured 1 day before implantation for IDS and BAI, a time point at which it might be speculated that anxiety and discomfort are transiently elevated owing to the upcoming procedure. The first FSAS assessment occurred 10 days after implantation, during a period when the risk of implantation-related complications is highest. However, no significant differences were found in FSAS scores between patients with and without an event in that time frame, supporting its validity as a baseline measure (Supplemental Table 6). Longitudinal assessment of shock-related anxiety revealed that patients without device-related events showed the most pronounced decline in fear of shock, whereas no significant changes were observed in those who did experience an event, which is in line with literature.^34^ This finding underscores the robustness of the FSAS measure in this population, but it should be noted that FSAS is used also for major complications although it is officially validated only for shocks.

Our cohort was predominantly male, which may limit generalizability given that previous studies suggest that female ICD patients often report higher levels of psychological distress.^21^^,^^40^^,^^41^ It should also be acknowledged that a statistically significant decrease in psychological distress does not necessarily correspond to a clinically meaningful improvement. Furthermore, our findings are based on specific anxiety and depression scales, and the value of alternative tools could be explored in future research. Finally, the integration of psychological, clinical, and device-related data enabled a comprehensive analysis of factors contributing to psychological distress in patients with ICDs. However, as an observational study, causal relationships cannot be established and findings should be interpreted as hypothesis generating.

## Conclusion

Shock-related anxiety, general anxiety, and depressive symptoms typically show a gradual decline over the 24 months after ICD implantation. Long-term psychological distress seems to be driven predominantly by pre-existing higher levels of depressive and anxiety symptoms rather than device therapy or complications. Early psychological screening and targeted support may help identify and assist high-risk patients.

## Disclosures

The authors have no conflicts of interest to disclose.

## Footnotes

Appendix Supplementary data associated with this article can be found in the online version at https://doi.org/10.1016/j.hroo.2026.04.004.
