# Supplementary material for: Device-related events do not alter long-term psychological distress after implantable cardioverter-defibrillator implantation: A 24-month prospective cohort study
Source: Heart Rhythm O2. 2026 Apr 11;7(8):1537–45. doi: 10.1016/j.hroo.2026.04.004 (PMC13523587; doi:10.1016/j.hroo.2026.04.004)
Supplement: Supplementary file 1 [file mmc1.docx]

**SUPPLEMENTS**

**Table S1:** scores for depression, anxiety, and fear of shock scores between baseline and 24M post implantation, compared using a Wilcoxon signed ranks test.

| **Total population (N=178)** | Baseline | 2M post-implantation | 12M post-implantation | 24M post-implantation | p-value  BL-2M | p-value 2M-12 | p-value 12M-24M | p-value BL-24M |
| --- | --- | --- | --- | --- | --- | --- | --- | --- |
| FSAS | 11 (10;14) | 10 (10;12) | 10 (10;12) | 10 (10;12) | **<0.001** | 0.140 | 0.735 | **<0.001** |
| IDS | 10 (7;18) | 8 (5;14) | 9.5 (5;17) | 9 (4;15) | **<0.001** | **<0.001** | 0.096 | **0.024** |
| BAI | 4 (2;9) | 3 (1;6) | 3 (1;7) | 3 (1;7) | **<0.001** | 0.620 | **0.032** | **0.045** |

Abbreviations: FSAS = Florida Shock Anxiety Scale, IDS = Inventory of Depressive Symptoms, BAI = Beck Anxiety Index.

**Table S2:** fear of shock scores compared between patients with and without particular events in the previous period (e.g. 12M values are based on the presence of an event during 2M-12M follow-up) using Mann Whitney U tests.

| **FSAS** | **Event** | **#** | **No event** | **#** | **p-value** |
| --- | --- | --- | --- | --- | --- |
| **Device related event** |  |  |  |  |  |
| **BL-2M (n=160)** | **12 [10;17.3]** | **26** | **10 [10;12]** | **152** | **0.010** |
| **2M-12M (n=143)** | 10 [10;12.5] | 27 | 10 [10;12] | 151 | 0.843 |
| **12M-24M (n=125)** | **12 [10;14.3]** | **21** | **10 [10;12]** | **157** | **0.037** |
| **BL-24M (n=125)** | 10 [10;13.5] | 59 | 10 [10;12] | 129 | 0.384 |
| **Any complication** |  |  |  |  |  |
| **BL-2M (n=160)** | 11.5 [10;14.8] | 17 | 10 [10;12] | 161 | 0.136 |
| **2M-12M (n=143)** | 10 [10;10] | 8 | 10 [10;12] | 170 | 0.534 |
| **12M-24M (n=125)** | 12 [10;12] | 9 | 10 [10;12] | 169 | 0.188 |
| **BL-24M (n=125)** | 10 [10;12] | 29 | 10 [10;12] | 149 | 0.978 |
| **Major complication** |  |  |  |  |  |
| **BL-2M (n=160)** | 11 [10;14] | 8 | 10 [10;12] | 170 | 0.412 |
| **2M-12M (n=143)** | 10 [10;10] | 7 | 10 [10;12] | 171 | 0.534 |
| **12M-24M (n=125)** | 12 [10;13.3] | 8 | 10 [10;12] | 170 | 0.287 |
| **BL-24M (n=125)** | 10 [10;12] | 20 | 10 [10;12] | 168 | 0.855 |
| **ICD therapy event** |  |  |  |  |  |
| **BL-2M (n=160)** | 12 [10;26] | 8 | 10 [10;12] | 170 | 0.138 |
| **2M-12M (n=143)** | 10 [10;13] | 20 | 10 [10;12] | 158 | 0.780 |
| **12M-24M (n=125)** | 12 [10;14] | 16 | 10 [10;12] | 162 | 0.108 |
| **BL-24M (n=125)** | 10 [10;13.5] | 43 | 10 [10;12] | 135 | 0.319 |
| **Appropriate ICD therapy** |  |  |  |  |  |
| **BL-2M (n=160)** | 11 [10;11] | 2 | 10 [10;12.3] | 176 | 0.994 |
| **2M-12M (n=143)** | 10 [10;13] | 15 | 10 [10;12] | 163 | 0.973 |
| **12M-24M (n=125)** | 11 [10;12] | 12 | 10 [10;12] | 166 | 0.903 |
| **BL-24M (n=125)** | 10 [10;13] | 29 | 10 [10;12] | 159 | 0.646 |
| **Inappropriate ICD therapy** |  |  |  |  |  |
| **BL-2M (n=160)** | 23 [10;27.5] | 6 | 10 [10;12] | 172 | 0.082 |
| **2M-12M (n=143)** | 11 [10;15] | 5 | 10 [10;12] | 173 | 0.642 |
| **12M-24M (n=125)** | 15 [14;15] | 4 | 10 [10;12] | 174 | 0.005 |
| **BL-24M (n=125)** | 11 [10;16] | 15 | 10 [10;12] | 163 | 0.165 |

Abbreviations: FSAS = Florida Shock Anxiety Scale, BL = baseline, ICD = implantable cardioverter defibrillator

**Table S3:** IDS scores compared between patients with and without particular events in the previous period (e.g. 12M values are based on the presence of an event during 2M-12M follow-up) using Mann Whitney U tests.

| **IDS** | **Event** | **#** | **No event** | **#** | **p-value** |
| --- | --- | --- | --- | --- | --- |
| **Device related event** |  |  |  |  |  |
| **BL-2M (n=160)** | 10.5 [6.3;18.5] | 26 | 8 [5;13] | 152 | 0.079 |
| **2M-12M (n=146)** | 7 [3;17.8] | 27 | 10 [5.3;17] | 151 | 0.188 |
| **12M-24M (n=60)** | 9 [3.8;15] | 21 | 9 [4;14.3] | 157 | 0.931 |
| **BL-24M (n=60)** | 10 [4;15] | 59 | 8 [4.5;14] | 129 | 0.981 |
| **Complication** |  |  |  |  |  |
| **BL-2M (n=160)** | 10 [5.3;18.3] | 17 | 8 [5;13] | 161 | 0.433 |
| **2M-12M (n=146)** | 20 [8;23] | 8 | 9 [5;17] | 170 | 0.254 |
| **12M-24M (n=60)** | 15 [3;15] | 9 | 9 [4;14] | 169 | 0.671 |
| **BL-24M (n=60)** | 14 [1.5;19.5] | 29 | 8.5 [4.3;13.8] | 149 | 0.728 |
| **Major complication** |  |  |  |  |  |
| **BL-2M (n=160)** | 15 [3;23] | 8 | 8 [5;13] | 170 | 0.415 |
| **2M-12M (n=146)** | 23 [5;23] | 7 | 9 [5;17] | 171 | 0.407 |
| **12M-24M (n=60)** | 9 [3;9] | 8 | 9 [4;14.3] | 170 | 0.869 |
| **BL-24M (n=60)** | 9 [0.8;24] | 20 | 9 [4.8;14] | 168 | 0.758 |
| **ICD therapy event** |  |  |  |  |  |
| **BL-2M (n=160)** | 9 [3;17] | 8 | 8 [5;13.5] | 170 | 0.738 |
| **2M-12M (n=146)** | 7 [3;17] | 20 | 10 [5;17] | 158 | 0.086 |
| **12M-24M (n=60)** | 14 [6.3;15] | 16 | 8.5 [4;14] | 162 | 0.389 |
| **BL-24M (n=60)** | 10 [4;15] | 43 | 8 [4.5;14.5] | 135 | 0.864 |
| **Appropriate ICD therapy** |  |  |  |  |  |
| **BL-2M (n=160)** | 5.5 [2;5.5] | 2 | 8 [5;14] | 176 | 0.370 |
| **2M-12M (n=146)** | 7 [3;17] | 15 | 10 [5;17] | 163 | 0.216 |
| **12M-24M (n=60)** | 14 [6.3;15] | 12 | 8.5 [4;14] | 166 | 0.389 |
| **BL-24M (n=60)** | 11 [4;15] | 29 | 8 [4;14] | 159 | 0.632 |
| **Inappropriate ICD therapy** |  |  |  |  |  |
| **BL-2M (n=160)** | 12 [5.5;21.5] | 6 | 8 [5;13] | 172 | 0.312 |
| **2M-12M (n=146)** | 5 [2.3;16.8] | 5 | 10 [5;17] | 173 | 0.232 |
| **12M-24M (n=60)** | N/a | 4 | 9 [4;14.8] | 174 | N/a |
| **BL-24M (n=60)** | 7 [1;10] | 15 | 9 [4.3;15] | 163 | 0.299 |

Abbreviations: IDS = Inventory of Depressive Symptoms, BL = baseline, ICD = implantable cardioverter defibrillator

**Table S4:** BAI scores compared between patients with and without particular events in the previous period (e.g. 12M values are based on the presence of an event during 2M-12M follow-up) using Mann Whitney U tests.

| **BAI** | **Event** | # | **No event** | **#** | **p-value** |
| --- | --- | --- | --- | --- | --- |
| **Device related event** |  |  |  |  |  |
| **BL-2M (n=159)** | 4 [2;8.5] | 26 | 2 [1;6] | 152 | 0.154 |
| **2M-12M (n=144)** | 2 [0.5;5.5] | 27 | 3 [1;7] | 151 | 0.243 |
| **12M-24M (n=132)** | 3.5 [1;7.3] | 21 | 3 [1;7.3] | 157 | 0.861 |
| **BL-24M (n=132)** | 3 [1;7] | 59 | 3.5 [1;7.8] | 129 | 0.703 |
| **Complication** |  |  |  |  |  |
| **BL-2M (n=159)** | 3.5 [0.3;6.8] | 17 | 2 [1;6] | 161 | 0.700 |
| **2M-12M (n=144)** | 5 [2;5] | 8 | 3 [1;7] | 170 | 0.311 |
| **12M-24M (n=132)** | 6 [0;9] | 9 | 3 [1;7] | 169 | 0.616 |
| **BL-24M (n=132)** | 3 [0.3;8.8] | 29 | 3 [1;7] | 149 | 0.888 |
| **Major complication** |  |  |  |  |  |
| **BL-2M (n=159)** | 3 [0;10] | 8 | 2.5 [1;6] | 170 | 0.865 |
| **2M-12M (n=144)** | 5 [2;5] | 7 | 3 [1;7] | 171 | 0.311 |
| **12M-24M (n=132)** | 5.5 [0;10.8] | 8 | 3 [1;7] | 170 | 0.725 |
| **BL-24M (n=132)** | 5 [0;10.3] | 20 | 3 [1;7] | 168 | 0.610 |
| **ICD therapy event** |  |  |  |  |  |
| **BL-2M (n=159)** | 2 [0;9] | 8 | 3 [1;6] | 170 | 0.909 |
| **2M-12M (n=144)** | 2 [0;5] | 20 | 3 [1;7] | 158 | 0.120 |
| **12M-24M (n=132)** | 4 [1;8] | 16 | 3 [1.7] | 162 | 0.678 |
| **BL-24M (n=132)** | 2 [0.8;7] | 43 | 4.5 [1;8] | 135 | 0.216 |
| **Appropriate ICD therapy** |  |  |  |  |  |
| **BL-2M (n=159)** | 1 [0;1] | 2 | 3 [1;6] | 176 | 0.274 |
| **2M-12M (n=144)** | 2 [1;5] | 15 | 3 [1;7] | 163 | 0.321 |
| **12M-24M (n=132)** | 3.5 [1;7.5] | 12 | 3 [1;7] | 166 | 0.897 |
| **BL-24M (n=132)** | 2.5 [1;5.5] | 29 | 3.5 [1;8] | 159 | 0.435 |
| **Inappropriate ICD therapy** |  |  |  |  |  |
| **BL-2M (n=159)** | 3 [1;18] | 6 | 2.5 [1;6] | 172 | 0.536 |
| **2M-12M (n=144)** | 1 [0;5] | 5 | 3 [1;7] | 173 | 0.197 |
| **12M-24M (n=132)** | 7 [2;7] | 4 | 3 [1;7] | 174 | 0.353 |
| **BL-24M (n=132)** | 2 [0;7] | 15 | 3 [1;8] | 163 | 0.326 |

Abbreviations: BAI = Beck Anxiety Index, BL = baseline, ICD = implantable cardioverter defibrillator

**Table S5:** FSAS, IDS, and BAI scores between patients with ICDs and patients with CRT-Ds at baseline and 24 months of follow-up.

|  | **ICD** | **CRT-D** | **p-value** |
| --- | --- | --- | --- |
|  | N=129 | N=49 |  |
| **Baseline** |  |  |  |
| FSAS | 11 [10-14] | 11 [10-14] | 0.641 |
| IDS | 10 [6-17] | 13.5 [8.3-22.5] | 0.129 |
| BAI | 4 [2-8.8] | 5.5 [2.3 - 13] | 0.560 |
| **24 months** |  |  |  |
| FSAS | 10 [10-12] | 10 [10-12.8] | 0.410 |
| IDS | 8 [4-13.8] | 10.5 [5.5-21.8] | 0.691 |
| BAI | 2.5 [1-7] | 5.5 [2-8.3] | 0.193 |

Abbreviations: FSAS = Florida Shock Anxiety Scale, IDS = Inventory of Depressive Symptoms, BAI = Beck Anxiety Index, ICD = implantable cardioverter defibrillator, CRT-D = cardiac resynchronization therapy – defibrillator

**Table S6:** FSAS score at 10 days compared between patients with and without an event between implantation and 10 days follow-up

| **FSAS (n=163)** | **Event in first 10 days** | **#** | **No event in first 10 days** | **#** | **p-value** |
| --- | --- | --- | --- | --- | --- |
| **Device related event** | 14 [10;17.8] | 16 | 11 [10;14] | 147 | 0.099 |
| **Complication** | 12 [10;16.5] | 12 | 11 [10;14] | 151 | 0.382 |
| **Major complication** | 15 [10;18] | 7 | 11 [10;14] | 156 | 0.120 |
| **ICD therapy event** | 17.5 [11.3;28.3] | 4 | 11 [10;14] | 159 | 0.090 |
| **Appropriate ICD therapy** | N/a | 0 | 11 [10;14] | 163 | N/a |
| **Inappropriate ICD therapy** | 17.5 [11.3;28.3] | 4 | 11 [10;14] | 159 | 0.090 |

Abbreviations: FSAS = Florida Shock Anxiety Scale, ICD = implantable cardioverter defibrillator

**Figure S1:** FSAS scores over time for patients with and without major complications (A1), appropriate ICD therapy (B1), and inappropriate ICD therapy (C1). In the right panel the FSAS scores from baseline to 24 months for patients with and without major complications (A2), appropriate ICD therapy (B2), and inappropriate ICD therapy (C2).
